# A dating app for extracellular adhesion proteins

**DOI:** 10.1016/j.xgen.2026.101247

**Published:** 2026-05-13

**Authors:** Casey D. Gailey, David M. Miller

**Affiliations:** 1 Department of Cell and Developmental Biology, Vanderbilt University School of Medicine, Nashville, TN 37240, USA; 2 Neuroscience Program, Vanderbilt University School of Medicine, Nashville, TN 37240, USA

## Abstract

Cell surfaces display dense arrays of extracellular proteins that interact with each other and with secreted cues. Nawrocka et al. deployed a biochemical screen to catalog these interactions at genome scale for the model organism *C. elegans* and identified new effectors of axon guidance, insulin signaling, growth factor biology, and neuronal connectivity.

## Main text

The metazoan body plan inherently depends on cell-to-cell communication involving proximal contacts among cell surface proteins as well as long range responses to secreted cues. Together, these interactions mediate a litany of cellular activities including adhesion, morphogenesis, migration, metabolism, and connectivity. Although directed approaches have successfully identified multiple instances of specific protein-protein interactions (PPIs) among cell surface proteins, the potential combinatorial landscape is vast. For example, the human genome encodes >500 of immunoglobulin superfamily (IgSF) proteins, each with the theoretical capacity to interact with multiple partners.^1^ In addition, weak interactions, which are characteristic of cell surface proteins, are typically not detected by biochemical pull-down methods. To address these challenges, Nawrocka et al. utilized a sensitive, high-throughput extracellular interaction assay (ECIA) to screen for thousands of potential PPIs in the model organism, *C. elegans*.^2^ In this approach, extracellular protein domains are fused to bait and prey complexes for expression in insect cells. Lysates were then tested for interaction in an enzymatically amplified microtiter plate assay. An earlier application of the ECIA strategy to *Drosophila melanogaster* tested 202 IgSF, fibronectin type III (FN3), and leucine-rich repeat (LRR) proteins to identify 102 interactions, 83 of which were previously unknown.^3^ In this new work, the authors exploit the limited number of paralogs in the compact *C. elegans* genome to encompass a more expansive list of 86 classes of protein domains including IgSF, FN3, LRR, EGF-like, TSP1, CUB, cadherin, lectins, GAIN, etc. In all, 379 cell surface domains and secreted proteins were tested to identify 159 previously unknown interactions.

**Figure 1:**
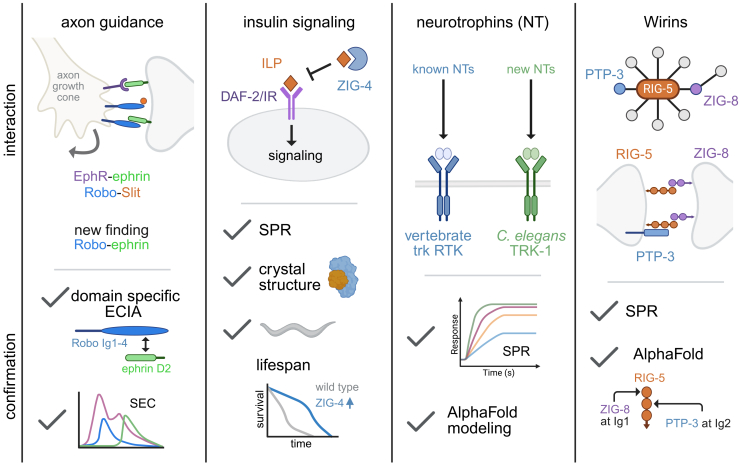
Validation of new protein-protein interactions The *C. elegans* extracellular interactome identified 159 previously unknown protein-protein interactions (PPIs). New PPIs were validated for axon guidance, insulin signaling, neurotrophin biology, and neural connectivity using size exclusion chromatography (SEC), surface plasmon resonance (SPR), structural models, and genetics. Created in BioRender: Miller, D. (2026), https://BioRender.com/0zj5147.

Novel classes of PPIs from the ECIA dataset were validated with additional experiments. For example, axonal trajectories in the nervous system depend on the specific interactions of receptors on the surface of axonal growth cones with external guidance cues (Figure 1). ECIA results confirmed canonical PPIs for the four families of core axonal guidance complexes composed of receptors vs. ligands: SAX-3/Robo vs. SLT-1/Slit, DCC/UNC-40 and UNC-5 vs. UNC-6/Netrin, VAB-1/EphR vs. EFN/Ephrin, and PLX/Plexin vs. MAB-20/semaphorin. Notably, previously unreported interactions were also detected. In one case, EFN-4, a member of the ephrin family of ligands, interacted with the Slit receptor SAX-3/Robo and with the semaphorin cue MAB-20. Size exclusion chromatography (SEC) detected a stable heteromeric complex between purified EFN-4 and the N-terminal Ig1-4 domains of the SAX-3 receptor that also interact with Slit/Slt-1, a finding that could be indicative of a novel mechanism for crosstalk between Slit and Ephrin guidance cues. Intriguingly, independent genetic data implicate MAB-20/semaphorin, SAX-3/Robo, and EFN-4/ephrin in common morphogenetic pathways in *C. elegans* that could be related to these newly found biochemical interactions.

In a second case, the authors investigated the unexpected interaction of insulin-like proteins (ILPs) with members of the ZIG family of secreted IgSFs (Figure 1). In nematodes, as in mammals, ILP signaling modulates metabolism and aging. *C. elegans* contains an expanded family of 40 ILPs but only one insulin receptor, DAF-2. ECIA screening revealed that specific ILPs interact with ZIG-2, ZIG-3, ZIG-4, and ZIG-5. ZIG proteins contain a tandem array of Ig domains and have been previously shown to modulate adhesion for neuronal circuit development and maintenance.^4^ Here, the authors utilize a powerful suite of biochemical, structural, and genetic methods to confirm that interaction with ZIG proteins also regulates ILP signaling. First, surface plasmon resonance (SPR) assays detected a high affinity dissociation constant (Kd = 56 pM) for interaction between INS-6/ILP and ZIG-4. Second, X-ray crystallography coupled with site-specific mutagenesis identified multiple interacting amino acid side chains that stabilize this complex. Intriguingly, the INS-6/ZIG-4 structure closely mimics that of a dipterid ZIG-like protein, ImpL2, bound to human or fly ILPs. Finally, over-expression of ZIG-4 antagonizes ILP-dependent pathways that regulate lifespan, a finding that also parallels earlier results with ImpL2 in *Drosophila*.^5^ The investigation of ILP interaction with ZIG proteins highlights a key strength of this work that exploits the ready application of *C. elegans* genetic tools to test the relevance of structural and biochemical data *in vivo*.

In a third case, the authors validated the interaction of potential ligands with neurotrophin and growth factor receptors. In mammals, neurotrophins (NGF, BDNF, NT-3, NT4/5) interact with Trk family receptor kinases to modulate key events in neuronal development and function (Figure 1). *C. elegans* encodes a single Trk receptor kinase, TRK-1, but no apparent sequence homologs of vertebrate neurotrophins. This work detected strong interactions between TRK-1 and potential functional neurotrophin homologs in *C. elegans* that contain the “cystine knot” motif, a characteristic arrangement of disulfide bonds that stabilizes neurotrophin structure. Alphafold predicts high-confidence structural models of the TRK-1 receptor and putative nematode neurotrophins that resemble mammalian TRK-neurotrophin complexes, thus suggesting that the neurotrophin signaling axis is evolutionarily ancient and can be productively investigated in *C. elegans*.

A final series of experiments surveyed PPIs with potential roles in circuit wiring, a key activity for cell adhesion proteins during neural development. In the first case, ECIA confirmed interaction between ZIG-8 and RIG-5, homologs of Wirin family proteins Dpr and DIP, respectively (Figure 1). Transcellular interactions between matched pairs of Dpr and DIP proteins have been proposed to induce neuron-specific synapses.^6^ Curiously, Wirins are anchored to the cell surface by a GPI linkage and do not include either transmembrane or intracellular domains for directing downstream clustering of synaptic components. The discovery in this work of multiple additional cell surface proteins that interact independently with either ZIG-8 or RIG-5 offers a potential mechanistic explanation for their roles in synaptic assembly. For example, RIG-5/DIP interacts with the extracellular domain of PTP-3, the sole *C. elegans* member of the LAR family of receptor tyrosine phosphatases (RTPTs). Importantly, RTPTs have been shown to promote clustering of presynaptic components at sites of transcellular interaction with post-synaptic adhesion proteins.^7^ Point mutations at predicted interaction sites in the Alphafold model of the RIG-5-PTP-3 complex validated the proposed structure by disrupting binding in SPR assays. These findings suggest a testable model in which the trans-synaptic RIG-5-ZIG-8 complex directs PTP-3 localization to nascent synapses. Indeed, recent work has shown that the RIG-5-ZIG-8 complex promotes assembly of the postsynaptic apparatus at specific pairs of *C. elegans* neurons via ZIG-8 interaction with an extracellular domain of the ACR-16 acetylcholine receptor.^8^ To consider the possible roles of other PPIs from their screen in circuit assembly, the authors relied on a gene expression atlas^9^ to map interacting protein pairs to synaptic partners in the wiring diagram of the *C. elegans* nervous system. Statistical criteria revealed a subset of candidate synaptogenic PPIs that can now be experimentally tested *in vivo*.

The results of this work provide a rich tableau of newly identified interactions among extracellular protein domains. Despite the impressive breadth of these findings, the design of the screening assay excludes other categories of important PPIs. For example, the extracellular regions of proteins with multiple transmembrane domains (e.g., GPCRs) are not represented. Complexes that depend on more than two interacting protein domains would also escape detection. In principle, a broader screen could be conducted *in silico*. Indeed, the authors show that AlphaFold scores for experimentally identified PPIs are robust in comparison to randomly selected partners. Although promising, this approach will likely require further refinement of computational tools to distinguish false positives from authentic interactions.^10^

## Acknowledgments

This work is supported by NIH grants (F31NS134292) to C.D.G. and (R01NS100547 and R01NS113559) to D.M.M.

## Declaration of interests

The authors declare no competing interests.
